# Relationship between comorbidity and health outcomes in patients with heart failure: a systematic review and meta-analysis

**DOI:** 10.1186/s12872-023-03527-x

**Published:** 2023-10-10

**Authors:** Kyoung Suk Lee, Da-In Park, Jihyang Lee, Oonjee Oh, Nayoung Kim, Gyumi Nam

**Affiliations:** 1 College of Nursing, The Research Institute of Nursing Science, Seoul National University, Seoul, Republic of Korea; 2 Department of Nursing, College of Life Science and Nano Technology, Hannam University, Daejeon, Republic of Korea; 3 Seoul National University Hospital, Seoul, Republic of Korea

**Keywords:** Heart failure, Comorbidity, Multimorbidity, Observational study, Systematic review, Meta-analysis

## Abstract

**Background:**

The prevalence of heart failure (HF) is expected to rise due to increased survivorship and life expectancy of patients with acute heart conditions. Patients with HF and other multiple comorbid conditions are likely to have poor health outcomes. This study aimed to assimilate the current body of knowledge and to provide the pooled effect of HF patients’ comorbid conditions on health outcomes.

**Methods:**

A systematic search was performed using MEDLINE, EMBASE and CINAHL databases. Observational studies evaluating the relationship between comorbid conditions and the health outcomes of HF were included. The pooled effect sizes of comorbidity on the identified health outcomes were calculated using a random effects model, and the heterogeneity was evaluated using I^2^ statistics.

**Results:**

A total of 42 studies were included in this review, and a meta-analysis was performed using the results of 39 studies. In the pooled analysis, the presence of a comorbid condition showed a significant pooled effect size in relation to the prognostic health outcomes: all-cause mortality (HR 1.31; 95% CI 1.18, 1.45), all-cause readmission (HR 1.16; 95% CI 1.09, 1.23), HF-related readmission (HR 1.13; 95% CI 1.05, 1.23), and non-HF-related readmission (HR 1.17; 95% CI 1.07, 1.27). Also, comorbidity was significantly associated with health-related quality of life and self-care confidence. Furthermore, we identified a total of 32 comorbid conditions from included studies. From these, 16 individual conditions were included in the meta-analyses, and we identified 10 comorbid conditions to have negative effects on overall prognostic outcomes: DM (HR 1.16, 95% CI 1.11, 1.22), COPD (HR 1.31, 95% CI 1.23, 1.39), CKD (HR 1.18, 95% CI 1.14, 1.23, stroke (HR 1.25, 95% CI 1.17, 1.31), IHD (HR 1.17, 95% CI 1.11, 1.23), anemia (HR 1.42, 95% CI 1.14, 1.78), cancer (HR 1.17, 95% CI 1.04, 1.32), atrial fibrillation (HR 1.25, 95% CI 1.01, 1.54), dementia (HR 1.19, 95% CI 1.03, 1.36) and depression (HR 1.17, 95% CI 1.04, 1.31).

**Conclusions:**

Comorbid conditions have significantly negative pooled effects on HF patient health outcomes, especially in regard to the prognostic health outcomes. Clinicians should carefully identify and manage these conditions when implementing HF interventions to improve prognostic outcomes.

**Supplementary Information:**

The online version contains supplementary material available at 10.1186/s12872-023-03527-x.

## Background

Heart failure (HF) is a complex clinical syndrome associated with immense burden and reduced quality of life. It has become a major public health challenge worldwide with a substantially high prevalence among older adults [1]. As we face the forthcoming super-aged society, a continuous rise in HF prevalence is expected especially in more developed countries with improved survival rates for acute heart conditions [2]. Although advanced medicine has allowed better management of acute-stage heart conditions, the health outcomes of patients with HF remain poor, largely due to the chronicity of the illness and the presence of comorbidity [3].

Comorbidity in HF has recently gained considerable attention owing to its negative impact on health outcomes. Presence of both cardiovascular and non-cardiovascular comorbidities are frequently observed in HF patients [4, 5]. Recent review studies have indicated that HF research has shifted from focusing on acute care regimens to managing pre-existing comorbid conditions that have negative impacts on health outcomes [5, 6]. More than 40% of HF patients live with at least five comorbid conditions, which account for up to 80% of hospital care needs [5, 7]. This population presents high rates of unplanned readmissions and complications throughout their remaining lifetimes, and these costly medical needs derive largely from their comorbid conditions [8]. Thus, addressing and managing comorbidities should be recognized to achieve better quality of life and patient outcomes. Recent review studies have indicated that non-cardiovascular comorbidity significantly increases the risk of poor prognostic outcomes in patients with HF [6, 9]. However, given that these studies limited their search to non-cardiovascular conditions and mortality, there remains a paucity of data to fully understand the outcomes of HF patients with various comorbidities including both non-cardiovascular and cardiovascular comorbidities. For clinicians to stratify patients with a higher possibility of a worse prognosis and to provide more effective care, it is vital to understand the effects of common HF comorbid conditions on different health outcomes.

## Methods

### Aims

The purpose of this review was to provide a comprehensive understanding of the magnitude of the association between comorbid conditions and health outcomes in patients with HF. The specific aims were to synthesize and assimilate the current body of knowledge on comorbidity and health outcomes in HF, to provide pooled effect sizes of identified comorbid conditions in HF on health outcome measures, and to assess the quality of the current body of evidence.

### Study design and search strategy

This study was conducted by systematically reviewing current literature and using meta-analysis. A focused systematic literature search was conducted to identify related studies published between January 2014 and September 2021 using MEDLINE, EMBASE, and CINAHL databases, with a guidance of a medical librarian. Search was restricted to studies published after 2013, considering that a previous meta-analysis study included articles published up to 2013 [6]. We also restricted the search language to English and study design to observational studies.

Relevant studies pertaining to the relationship between comorbid conditions and outcomes in patients with HF were identified using the following relevant search terms with abbreviations and truncation: “heart failure” and “comorbidity.” Also, “multimorbidity” and its variations were combined with “comorbidity” and its variations using the “OR” operator. We did not set limitations for the outcome measures to synthesize the most literature possible. After reviewing the full texts of the articles, we further hand-searched the reference lists of each identified article.

The meta-analysis protocol was pre-registered with PROSPERO prior to the literature search process (CRD42020220021) in accordance with the recommendations of the Preferred Items for Systematic Reviews and Meta-Analyses (PRISMA) 2020 statement [10].

### Study selection

Studies were included in the analyses if they met the following criteria: conducted with an adult population 18 years or above with HF diagnosis; reported original results (e.g., not a literature review, editorial, or case study); reported the relationships between health outcomes and comorbidities, which were defined as clinical diagnoses based on medical records, administration codes, or self-report (e.g., not a condition determined by questionnaires for screening, such as the presence of clinically significant depressive symptoms based on the Patient Health Questionnaire-9); and reported relevant statistical values. To calculate effect sizes for each health outcome, we excluded studies that reported composite outcomes, such as combined results of morbidity and hospital readmissions.

### Data abstraction

Three reviewers (JL, OO, NK) independently screened all articles identified in the literature search process and retrieved full-text publications of all potentially eligible articles. The full-text articles were independently assessed by four reviewers (KSL, DP, JL, OO) for inclusion eligibility. Any discrepancies were resolved by discussion. A structured template was predefined and designed prior to the literature search process to ensure consistency of data extraction. Data were extracted by four reviewers (DP, JL, OO, GN) using the pre-defined template. After data extraction, the accuracy of all data was independently verified by two reviewers (KSL, DP).

### Quality appraisal

The Risk of Bias Assessment tool for Non-randomized Studies (RoBANS) (11) was used to assess the quality of each included study based on the risk of bias in six domains: the selection of participants, confounding variables, measurement of exposure, blinding of outcome assessment, incomplete outcome data, and selective outcome reporting. These six domains were judged to be “low,” “high,” or “unclear.” Three reviewers (KSL, DP, JL) first rated the quality of three articles independently using the RoBANS, and any disagreement was resolved through discussion and/or consulting with a fourth reviewer (OO). The quality of the remaining articles was reviewed by the three reviewers as well.

### Statistical analyses

Based on a predefined study protocol, we conducted a meta-analysis of the included studies. We used the RevMan software (version 5.4.1) and the CMA software (version 3.3.070) to perform all statistical analyses and to derive the forest plots. A meta-analysis was performed only when at least two studies provided data for each outcome of interest. We extracted adjusted hazard ratios (HR) or odds ratios (OR) with the corresponding 95% confidence intervals (CI) from the identified studies and used the inverse-variance method to calculate the overall effect size with 95% CI. HRs are considered a common measure of the association between comorbidities and HF prognostic outcomes with both ORs and RRs considered equivalent to HRs, given that the ORs and RRs provide similar estimates of risk when the outcome is rare [12]. If one study reported multiple adjusted HR or OR values for the same outcome measure, we calculated the pooled effect size that represented the outcome of the study.

The health outcome values were categorized into patient-reported and prognostic outcomes. The patient-reported outcomes included health-related quality of life (HRQoL) and self-care. The prognostic outcomes included mortality, hospital readmission and a longer hospital stay (> 4 days). If possible, we further categorized the outcome results based on the follow-up period of each study. In accordance with recent multicenter cohort and meta-analysis studies, we used the 1-year mark as the dividing point between short-term and long- term periods [13–15]. We then synthesized the pooled effect sizes of the most common comorbid conditions on health outcomes.

Considering the observational design of the studies and methodological differences, the random effects model was used [16]. Statistical heterogeneity was assessed using I^2^ statistics, where values of 25%, 50%, and 75% were considered as cut-off points for low, moderate, and high degrees of heterogeneity, respectively [17]. A *p*-value of < 0.05 was considered statistically significant. All studies in each forest plot were organized in alphabetical order. We planned to perform a meta-regression using clinical characteristics (e.g., New York Heart Association functional classification, and ejection fraction). However, not all studies reported the exact values, so we could not conduct the meta-regression. Publication bias was assessed using Begg and Egger’s test and funnel plot, and a *p*-value of > 0.05 was considered statistical evidence for absence of publication bias (18). To determine the effect of publication bias on the robustness of the synthesized analysis, Duval and Tweedie’s trim and fill method was used [18].

## Results

After duplicate removal and title and abstract review, 98 articles were selected for full-text reviews. Additional 29 articles were retrieved during hand-search process. After independent full-text reviews and discussions, a total of 42 studies were eligible for the final inclusion. From the 42 identified studies, a meta-analysis was performed using 39 studies (Fig S1).

### Narrative review of the included studies

The included 42 studies represented 2,814,442 HF patients recruited from inpatient [19–42], outpatient [43–53] or both settings [54–60] (Table S1).

The studies included mainly older adults with mean age ranging between 56 and 85 years. Included studies evaluated patients with both non-cardiovascular and cardiovascular comorbid conditions, yielding up to 32 individual conditions. The most commonly identified co-existing non-cardiovascular chronic conditions were diabetes mellitus (DM) [19–21, 24, 27, 29, 31–33, 35–37, 39, 42, 43, 46, 48, 50–52, 54, 56–59], chronic obstructive pulmonary disease (COPD) [27, 29, 31, 32, 36, 39–42, 44, 48, 51, 52, 56–60] and chronic kidney disease (CKD) [20, 22, 27, 29, 31, 36, 42, 46, 48–50, 52, 56–58], while the most frequently identified cardiovascular conditions were stroke [22, 23, 27, 29, 30, 32, 42, 51, 52, 54, 56–58], hypertension (HTN) [20, 29, 32, 36, 37, 50, 51, 54, 56, 58] and ischemic heart disease (IHD) [27, 31, 32, 36, 37, 39, 51, 54]. Some of the included studies defined comorbidity using the Charlson Comorbidity Index [23, 25, 26, 28, 32–34, 47, 51, 53, 55] or the total number of existing conditions [38, 45]. It should be noted that some of our included studies had exclusion criteria for patients who were diagnosed and receiving treatment for chronic conditions such as CKD [26], thyroid conditions [21], respiratory conditions [44] or psychological conditions [23].

The patient-reported outcomes included HRQoL [40, 47, 57] and self-care [23, 43, 50, 53, 55]. The prognostic outcomes included in-hospital mortality [22, 24, 27, 30, 33, 34, 41, 58], all-cause mortality [19, 20, 25–28, 32, 37, 38, 44, 48, 49, 51, 52, 56, 58–60], all-cause readmission [28, 29, 31, 33, 35, 38, 39, 42, 45, 46, 56, 59], HF-related readmission [21, 29, 36, 38, 52, 54, 58], non-HF-related readmission [29, 54] and longer hospital stay [24, 33]. Further evaluation of the studies investigating all-cause mortality identified eight studies that reported the effect of comorbidities on short-term mortality [25, 27, 28, 32, 38, 52, 58, 60], and eleven reporting on long-term mortality [19, 20, 26, 32, 37, 44, 48, 49, 51, 56, 59].

### Quality appraisal of included studies

Most included studies showed a low risk of bias in the selection of participants, confounding variables, measurement of exposure, blinding of outcome assessments, and incomplete outcome data domains. However, except for two studies that clearly reported the presence of the study protocol [28, 40], the majority of studies had unclear or a high risk of bias in selective reporting bias [20, 21, 23–27, 30, 33–36, 38, 39, 41, 43–45, 47, 49, 50, 52, 54–63] (Fig S2). Publication bias was, first, assessed by using the funnel plots of standard error with logit effect size. Examination of the funnel plots did not suggest publication bias as no asymmetry was observed. Additional Egger’s regression tests were performed to confirm the absence of publication bias for included studies for calculation of the overall effect size of comorbid conditions on prognostic outcomes (*p* = 0.29, two-tailed). Lastly, the trim and fill method revealed that no study was trimmed. As for studies that were included for other outcomes, the Egger’s test was not used due to the small number. However, examination of the funnel plots indicated an absence of publication bias.

### Quantitative analyses

Only a few studies were available for meta-analysis of patient-reported health outcomes. Although four studies investigated the relationship between comorbidity and HRQoL, only two were sufficient for the meta-analysis because of data limitations and differences in the questionnaires used to measure the level of HRQoL. The two studies used overall scores of the Kansas City Cardiomyopathy Questionnaire (KCCQ) [40, 57]. For self-care, studies that used Self-care of Heart Failure Index (SCHFI) were included in the meta-analysis [23, 43, 53]. We were able to perform the meta-analysis for three domains of the SCHFI: confidence, maintenance and management.

A total of 34 primary studies were pooled to calculate the overall effect size of comorbid conditions on prognostic outcomes: in-hospital mortality [22, 24, 27, 30, 33, 34, 41, 58], all-cause mortality [19, 20, 25–28, 37, 38, 44, 48, 49, 51, 52, 56, 58–60], all-cause readmission [28, 29, 31, 33, 35, 38, 39, 42, 45, 46, 56, 59], HF-related readmission [21, 29, 36, 38, 52, 54, 58], non-HF-related readmission [29, 54] and delayed length of hospital stay [24, 33]. Among the prognostic outcome measures, all-cause mortality was the only measure with sufficient data for the meta-analysis based on the follow-up periods. Eight studies were pooled to calculate the effect size of comorbid conditions on short-term all-cause mortality [25, 27, 28, 32, 38, 52, 58, 60], and 11 studies were pooled for long-term all-cause mortality [19, 20, 26, 32, 37, 44, 48, 49, 51, 56, 59].

#### Overall effects of comorbidity on HF outcome measures

**Fig. 1:**
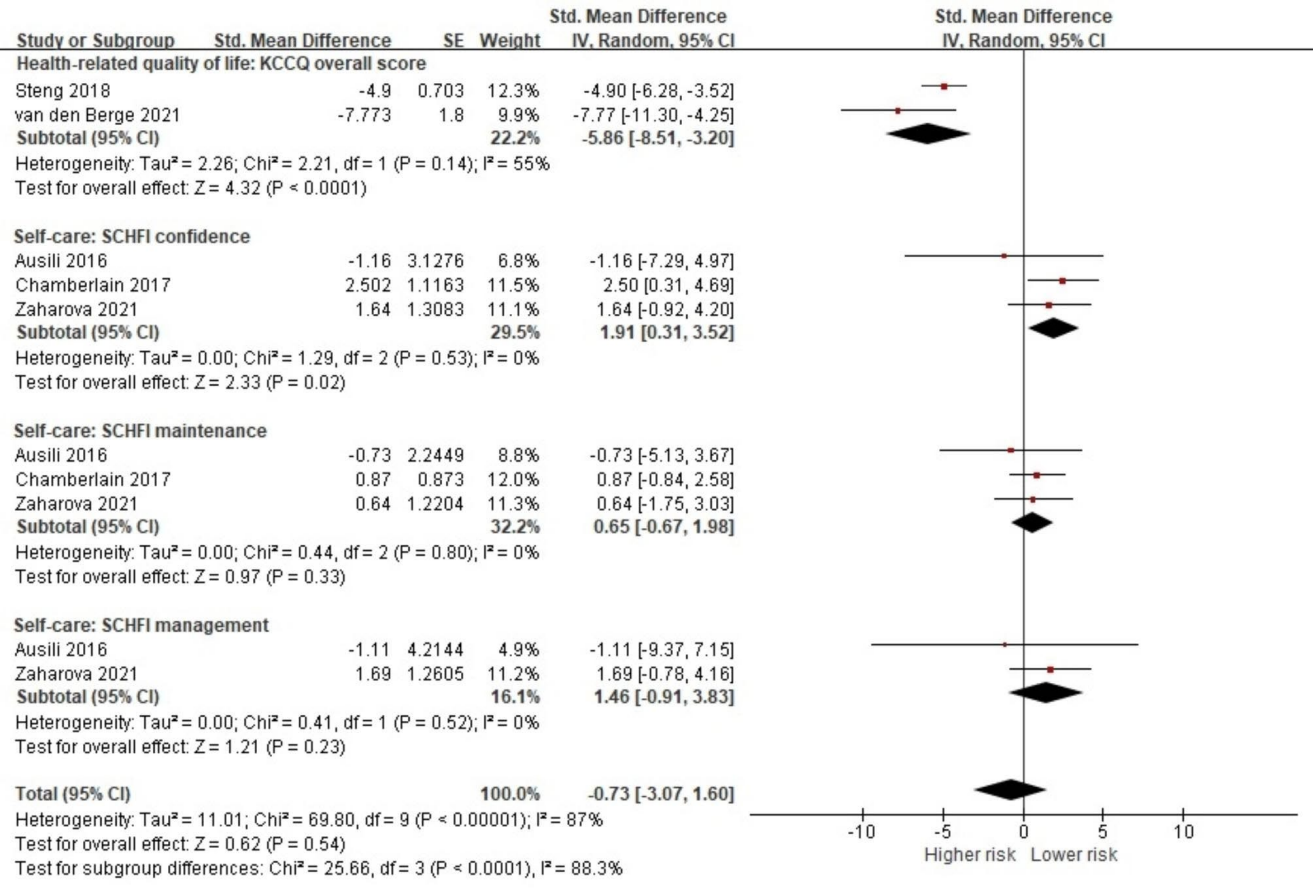
Forest plot of the pooled analysis evaluating the effect of comorbidities on overall patient-reported outcomes in heart failure patients

From included studies, only five studies were available to pool the data on patient-reported health outcome measures [23, 40, 43, 53, 57]. Although the presence of comorbidity did not show significant pooled effect size on overall patient-reported outcomes, significance was noted for the HRQoL measured by KCCQ overall score (standardized mean difference (SMD) -5.86, 95% CI -8.51, -3.20, *p <* 0.001) and the self-care confidence domain based on the SCHFI (SMD 1.91, 95% CI 0.31, 3.52, *p* = 0.02) [23, 43, 53] (Fig. 1).

**Fig. 2:**
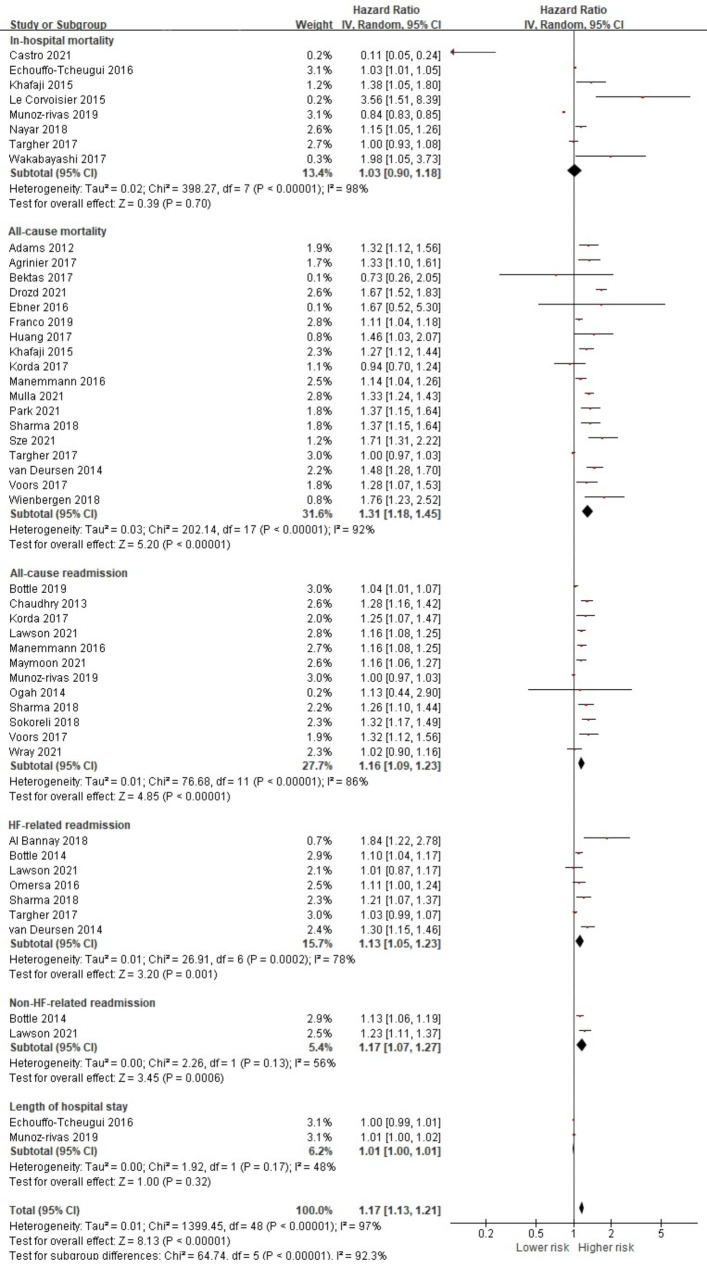
Forest plot of the pooled analysis evaluating the effect of comorbidities on overall prognostic health outcomes in patients with heart failure

On the other hand, the pooled analysis showed that the presence of any comorbid condition was associated with a significantly higher risk of overall prognostic health outcome with HF 1.17 (95% CI 1.13, 1.21, *p* < 0.001) (Fig. 2). The pooled analysis of 18 studies [19, 20, 25–28, 32, 37, 38, 44, 48, 49, 51, 52, 56, 58–60] indicated that HF patients with comorbidity had a statistically higher risk of all-cause mortality (HR 1.31, 95% CI 1.18, 1.45, *p* < 0.001). In addition, the presence of comorbidity significantly increased the risk of all-cause readmission (HR 1.16, 95% CI 1.09, 1.23, *p* < 0.001) [28, 29, 31, 33, 35, 38, 39, 42, 45, 46, 56, 59], HF-related readmission (HR 1.13, 95% CI 1.05, 1.23, *p* = 0.001) [21, 29, 36, 38, 52, 54, 58] and non-HF-related readmission (HR 1.17, 95% CI 1.07, 1.27, *p <* 0.001) [29, 54]. However, no significance was noted for in-hospital mortality and longer length of hospital stay (Fig. 2). When we further investigated the effects of coexisting conditions on all-cause mortality based on follow-up periods, we found a significant association for both short-term (HR 1.23, 95% CI 1.09, 1.38, *p* < 0.001) [25, 27, 28, 32, 38, 52, 58, 60] and long-term risks (HR 1.35, 95% CI 1.21, 1.50, *p* < 0.001) [19, 20, 26, 32, 37, 44, 48, 49, 51, 56, 59] (Fig S3).

#### Associated comorbid conditions

Although we identified various comorbid conditions, the following 16 individual conditions were identified for the meta-analysis: DM, COPD, CKD, stroke, HTN, IHD, anemia, cancer, atrial fibrillation, dementia, obesity, depression, arrhythmia, arthritis, asthma and valvular disease.

**Fig. 3:**
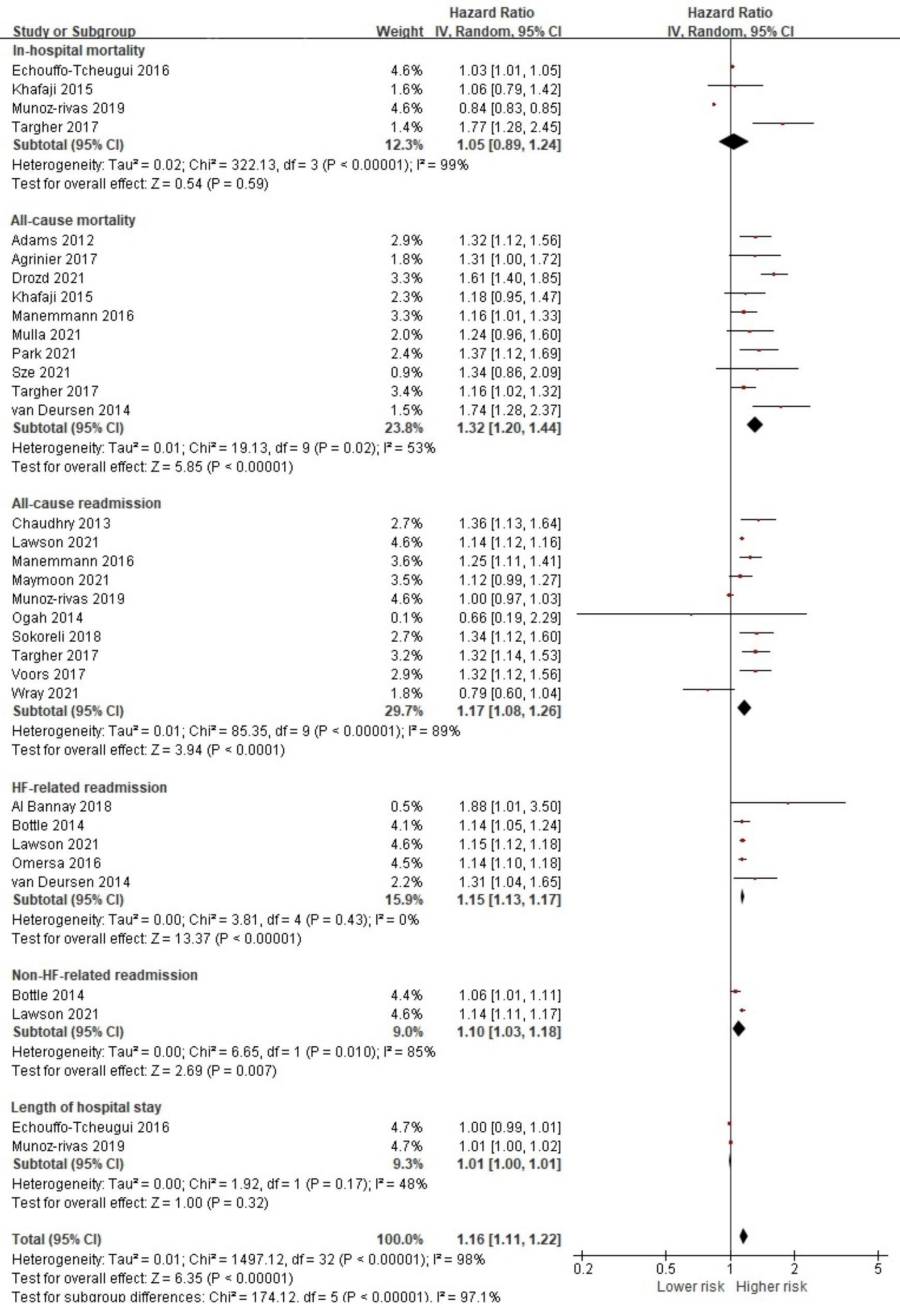
Forest plot of the pooled analysis evaluating the effect of diabetes mellitus on health outcomes in patients with heart failure

From 25 studies investigating DM, data from 22 studies were extracted to conduct a quantitative synthesis of the association between DM and the prognostic outcome measures [19–21, 24, 27, 29, 31–33, 35–37, 39, 42, 46, 48, 51, 52, 54, 56, 58, 59]. Diagnosis of DM was reported in 947,154 (34.8%) HF patients. Although the study results were highly heterogeneous (I^2^ = 98.0%) and, thus, should be interpreted with caution, the pooled effect size indicated that the presence of DM was adversely associated with the overall prognostic outcomes (HR 1.16, 95% CI 1.11, 1.22, *p* < 0.001) (Fig. 3). Our analysis indicated that, similar to the overall effect of any comorbid condition, HF patients with DM had an increased risk of all-cause mortality (HR 1.32, 95% CI 1.20, 1.44, *p* < 0.001), all-cause readmission (HR 1.17, 95% CI 1.08, 1.26, *p* < 0.001), HF-related readmission (HR 1.15, 95% CI 1.13, 1.17, *p* < 0.001) and non-HF-related readmission (HR 1.10, 95% CI 1.03, 1.18, *p* = 0.007). For all-cause mortality, further analysis was conducted using data from four studies with short-term follow-up [27, 32, 52, 58] and seven studies with long-term follow-up [19, 20, 32, 37, 48, 51, 56]. The presence of DM in HF significantly increased both short-term (HR 1.24, 95% CI 1.04, 1.46, *p* = 0.01) and long-term all-cause mortality (HR 1.35, 95% CI 1.23, 1.49, *p* < 0.001) (Fig S4).

**Fig. 4:**
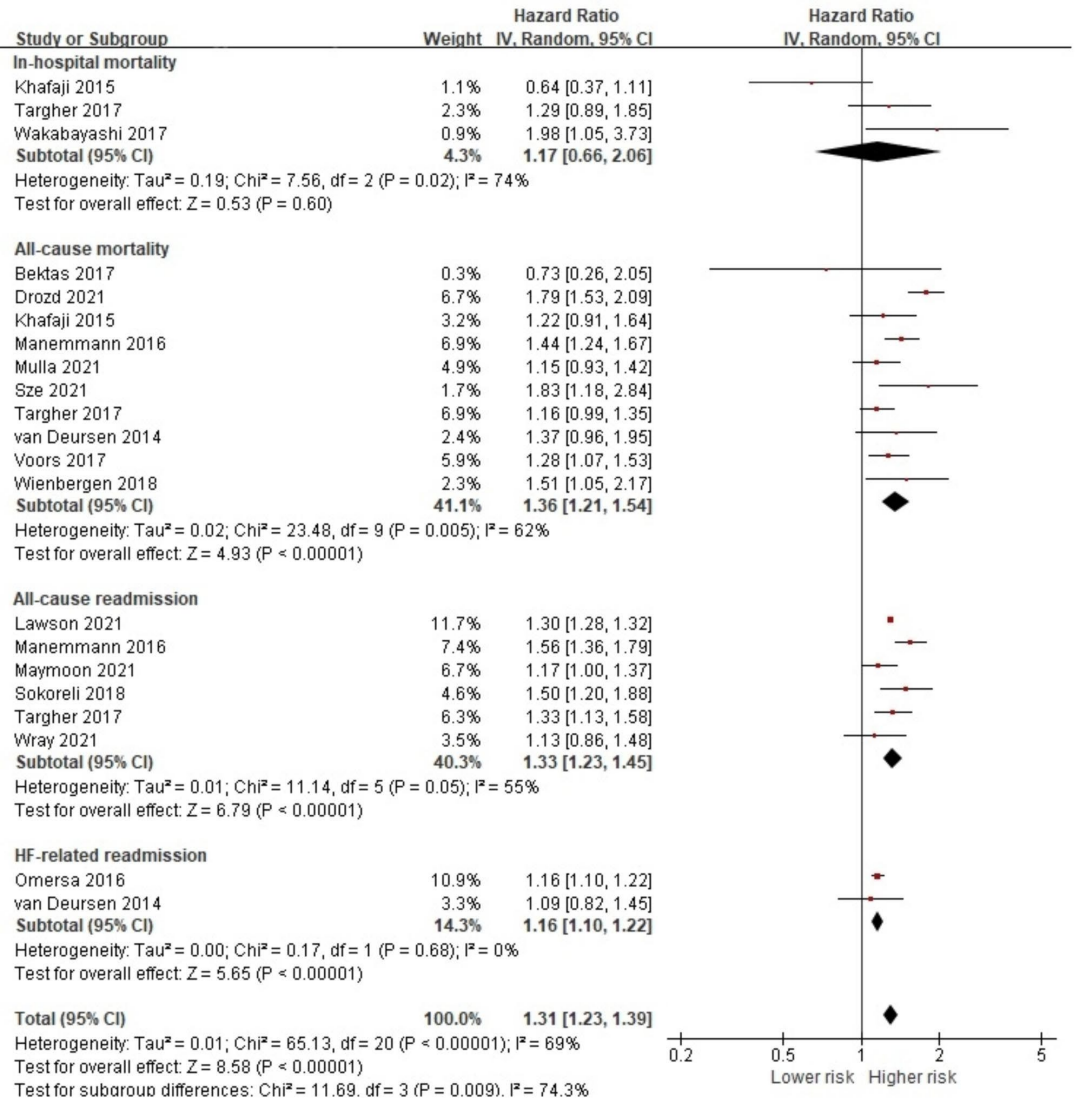
Forest plot of the pooled analysis evaluating the effect of chronic obstructive pulmonary disease on health outcomes in heart failure patients

From 18 studies that assessed COPD in HF, 16 reported the associations of COPD and in-hospital mortality [27, 41, 58], all-cause mortality [27, 32, 44, 48, 51, 52, 56, 58–60], all-cause readmission [29, 31, 39, 42, 56, 58] and HF-related readmission [36, 40]. Of the 783,940 HF patients, 108,488 (13.8%) were diagnosed with COPD, which increased the risk of poor overall prognostic health outcomes (HR 1.31, 95% CI 1.23, 1.39, *p* < 0.001) (Fig. 4). Significant risks were noted for all-cause mortality, all-cause readmission and HF-related readmission (HR 1.36, 95% CI 1.21, 1.54; HR 1.33, 95% CI 1.23, 1.45; HR 1.16, 95% CI 1.10, 1.22, respectively). Additional analysis also showed that a COPD condition was statistically significantly associated with both short-term (HR 1.22, 95% CI 1.09, 1.37, *p* < 0.001) [27, 32, 52, 58, 60] and long-term all-cause mortality risks (HR 1.43, 95% CI 1.20, 1.69, *p* < 0.001) [32, 44, 48, 51, 56, 59] (Fig S5).

**Fig. 5:**
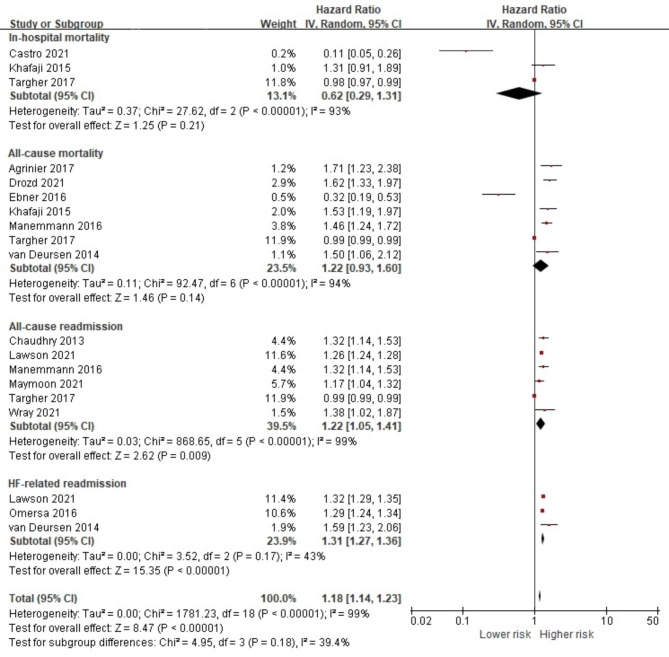
Forest plot of the pooled analysis evaluating the effect of chronic kidney disease on health outcomes in heart failure patients

The presence of CKD was observed in 122,715 (15.8%) patients in 13 included studies [20, 22, 27, 29, 31, 36, 42, 46, 48, 49, 52, 56, 58]. Although two additional studies also included HF patients with an underlying CKD condition, they did not provide sufficient data for the meta-analysis [50, 57]. We also pooled the data in-hospital mortality [22, 27, 58], all-cause mortality [20, 27, 48, 49, 52, 56, 58], all-cause readmission [29, 31, 42, 46, 56, 58], and HF-related readmission [29, 36, 52]. A higher risk of a poor overall prognosis outcome was observed with CKD (HR 1.18, 95% CI 1.14, 1.23, *p* < 0.001). HF patients with CKD showed a poorer prognosis in all-cause readmission (HR 1.22, 95% CI 1.05, 1.41, *p* = 0.009) and HF-related readmission (HR 1.31, 95% CI 1.27, 1.36, *p* < 0.001), but no significant relationship was noted for in-hospital mortality and all-cause mortality (Fig. 5 and S6).

**Fig. 6:**
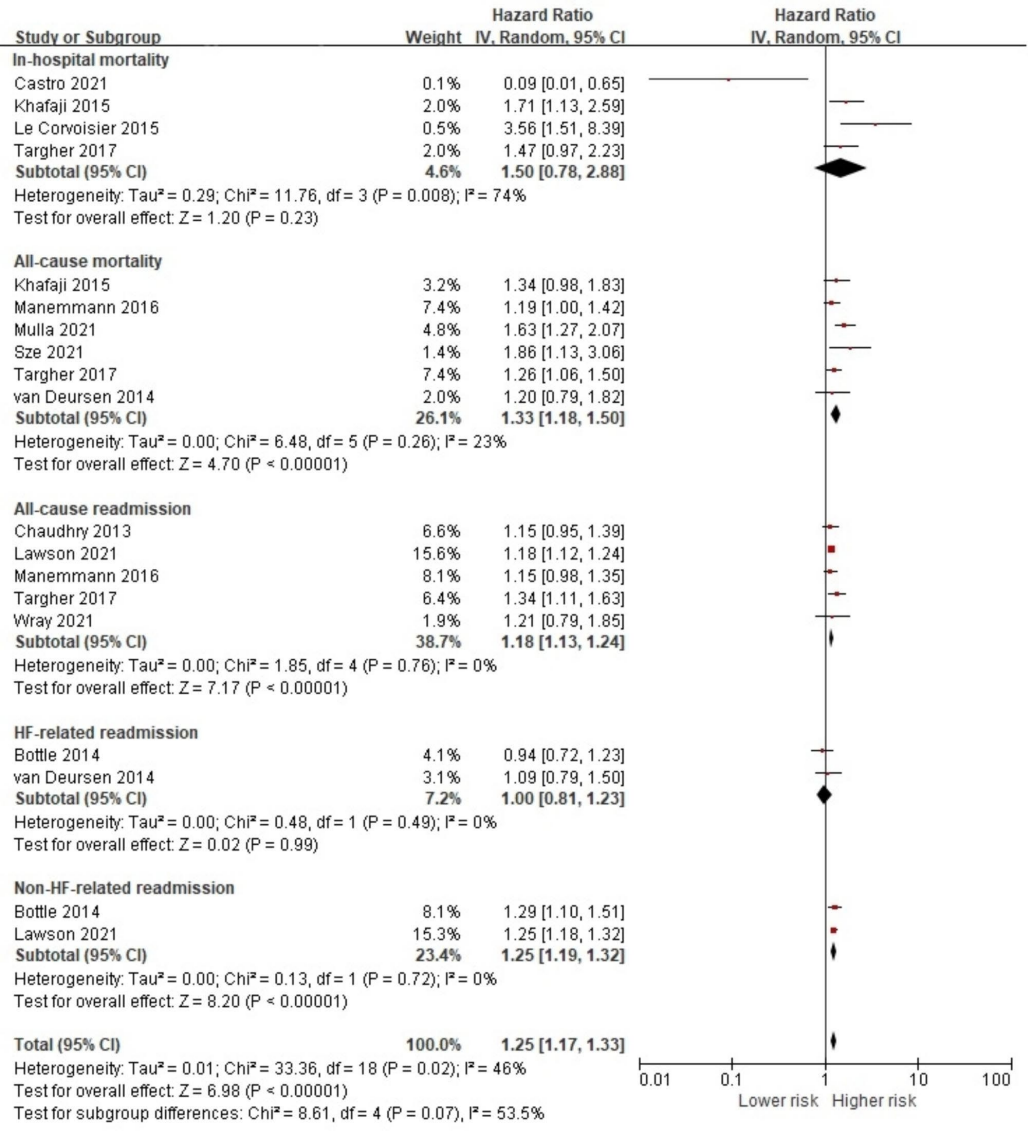
Forest plot of the pooled analysis evaluating the effect of stroke on health outcomes in heart failure patients

A history of stroke, also reported as cerebrovascular accident, was indicated in 44,998 (5.6%) patients in 13 studies. Because only one of the 13 studies reported patient-reported outcome measures [57], only 12 studies were pooled to calculate the effect size on the prognostic health outcomes: in-hospital mortality [22, 27, 30, 58], all-cause mortality [27, 32, 51, 52, 56, 58], all-cause readmission [29, 42, 46, 56, 58], HF-related readmission [52, 54] and non-HF-related readmission [29, 54]. A history of stroke had a significant pooled effect on a poor overall prognosis (HR 1.25, 95% CI 1.17, 1.33, *p* < 0.001), specifically on all-cause mortality (HR 1.33, 95% CI 1.18, 1.50, *p* < 0.001), all-cause readmission (HR 1.18, 95% CI 1.13, 1.24, *p* < 0.001) and non-HF-related readmission risks (HR 1.25, 95% CI 1.19, 1.32, *p* < 0.001) (Fig. 6). When we conducted a meta-analysis on all-cause mortality based on the follow-up periods, both short-term (HR 1.31, 95% CI 1.15, 1.51, *p* < 0.001), and long-term all-cause mortality risks (HR 1.40, 95% CI 1.10, 1.79, *p* < 0.01) significantly increased with a history of stroke (Fig S7).

**Fig. 7:**
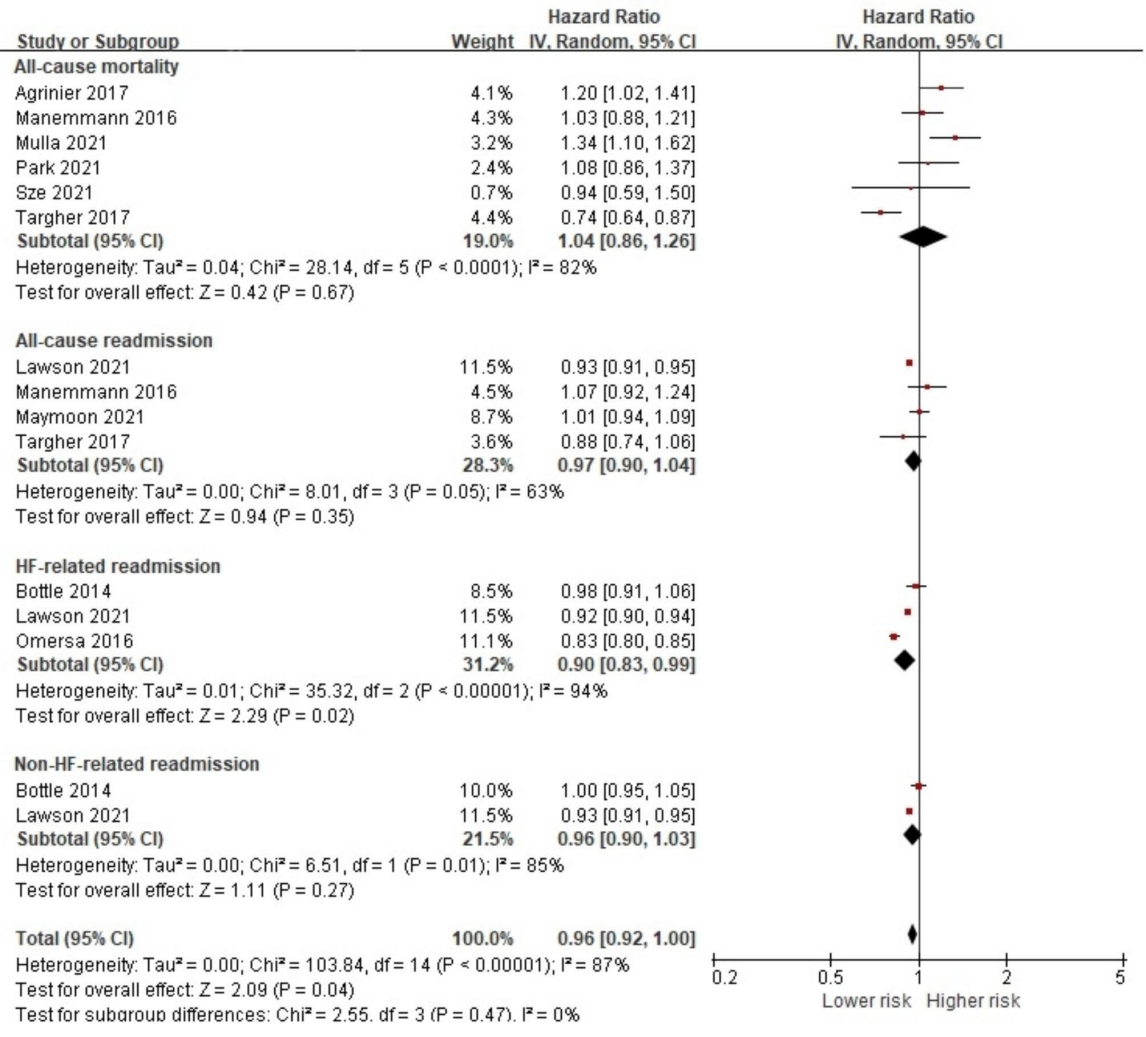
Forest plot of the pooled analysis evaluating the effect of hypertension on health outcomes in heart failure patients

The presence of HTN was observed in 386,154 (46.7%) patients in 11 studies, but data were extracted from 10 studies reporting the association of HTN with all-cause mortality [20, 32, 37, 51, 56, 58], all-cause readmission [29, 31, 56, 58], HF-related readmission [29, 36, 54] and non-HF-related readmission [29, 54]. The presence of HTN did not have a significant effect on the overall prognosis outcome in HF patients. In addition, it did not have a significant association with all-cause mortality, all-cause readmission, HF-related readmission, and short-term all-cause mortality risk (Fig. 7 and S8). However, HF patients with an HTN diagnosis showed a significantly higher long-term all-cause mortality (HR 1.16, 95% CI 1.01, 1.32, *p* = 0.03) but lower risk of HF-related readmission (HR 0.90, 95% CI 0.83, 0.99, *p* = 0.02).

**Fig. 8:**
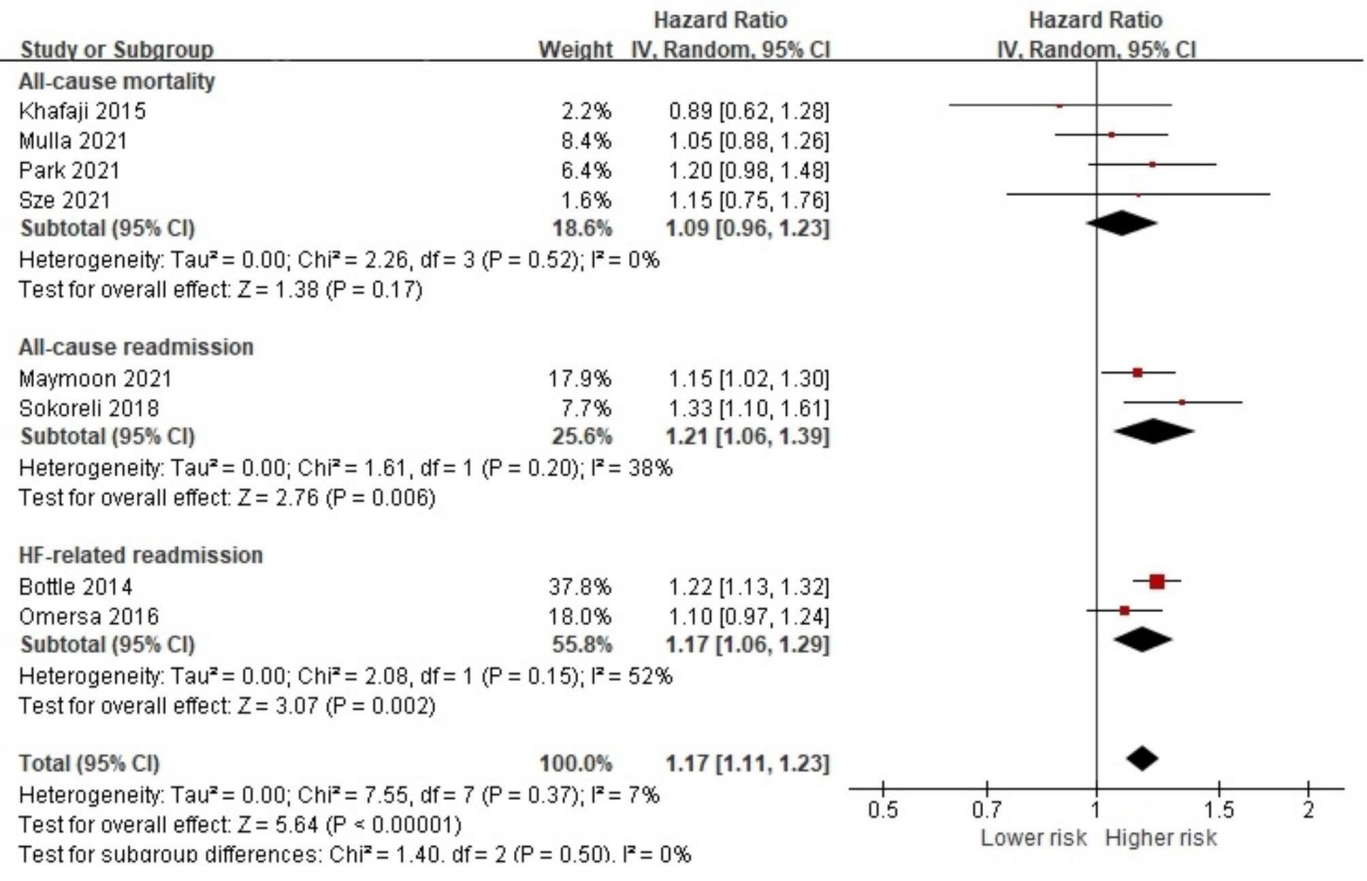
Forest plot of the pooled analysis evaluating the effect of ischemic heart disease on health outcomes in heart failure patients

We identified 42,773 (34.6%) HF patients with IHD in eight studies that observed the impact of IHD on all-cause mortality [27, 32, 37, 51], all-cause readmission [31, 39] and HF-related readmission [36, 54]. IHD in HF patients increased the risk of the overall prognostic outcome (HR 1.17, 95% CI 1.11, 1.23, *p* < 0.001), especially in regard to all-cause readmission (HR 1.21, 95% CI 1.06, 1.39, *p* = 0.006) and HF-related readmission (HR 1.17, 95% CI 1.06, 1.29, *p* = 0.002) (Fig. 8). However, it should be noted that only two studies were available for the meta-analysis for all-cause readmission and HF-related readmission. No statistical significance was noted for all-cause mortality and all-cause mortality based on the follow-up period (Fig S9).

A history of anemia (HR 1.42, 95% CI 1.14, 1.78, *p* < 0.01) [28, 29, 31, 49, 51, 52, 60], cancer (HR 1.17, 95% CI 1.04, 1.32, *p* = 0.01) [29, 36, 39, 42, 56, 60], atrial fibrillation (HR 1.25, 95% CI 1.01, 1.54, *p* = 0.04) [31, 32, 35, 37], dementia (HR 1.19, 95% CI 1.03, 1.36, *p* = 0.02) [28, 29, 42, 51, 54, 56] and depression (HR 1.17, 95% CI 1.04, 1.31, *p* < 0.01) [29, 51, 56] were found to have a significant pooled effect on the overall prognostic outcomes in HF (Figures S10 to S15). However, no significant effect of obesity on prognostic outcomes was noted (HR 0.99, 95% CI 0.98, 1.00) [31, 49, 58]. Few studies reported HRs of arrhythmia [42, 56], arthritis [29, 56], asthma [29, 42, 56] and valvular disease [31, 42] for all-cause readmission. Of these conditions, HF patients who were diagnosed with arrhythmia and arthritis had an increased risk of all-cause readmission (HR 1.18, 95% CI 1.06, 1.31; HR 1.16, 95% CI 1.11, 1.20, respectively) (Fig S16).

## Discussion

A total of 42 studies were included in this review, and the results of 39 studies were pooled to perform meta-analyses to calculate the overall effect size of presence of comorbidities on both patient-reported and prognostic health outcomes and to calculate the effect size of individual comorbid condition on health outcomes as well. Due to the small number of studies that evaluated patient-reported outcomes, no significance was noted for the pooled effect size of comorbidities in HF on overall patient-reported outcomes. However, we found that the presence of comorbid conditions to have significant pooled effect on overall prognostic health outcomes. Further evaluation indicated that the presence of comorbidities had a significantly negative effect on all-cause mortality with both the short- and long-term follow-up periods. Although limited data was available to pool effect size of individual comorbid condition on patient-reported outcomes, we identified DM, COPD, CKD, stroke, IHD, anemia, cancer, atrial fibrillation, dementia, and depression to have significant pooled effect on poorer prognostic health outcomes. In addition, arrhythmia and arthritis were found to increase the risk of all-cause readmission.

Although the effect size on overall patient-reported outcomes was not significant, our quantitative analysis showed that the co-occurrence of HF and other chronic conditions is likely to result in poor HRQoL and self-care confidence levels. However, it should be noted that only a few of the included studies have measured patient-reported outcomes; therefore, our results may underrepresent patient-reported outcomes. Patient-reported outcomes include diverse factors that can be measured using various instruments. Because when it comes to patient-reported outcomes, such as HRQoL and self-care, similar outcomes can be measured with different instruments. A similar issue was raised in a previous scoping review, where the authors attempted to pool the effect of comorbidities on quality of life but were unable to do so due to wide variations in assessment methods between studies [64].

A previous meta-analytic study has also indicated that non-cardiovascular comorbidities in HF patients were associated with all-cause mortality (6). However, given that Rushton’s review was only able to synthesize studies on mortality, our results further broaden the understanding of the effect of HF comorbidity on patient prognosis. In addition, to the best of our knowledge, no meta-analysis has been conducted to interpret the pooled effect of comorbidities on HF patient outcomes based on the follow-up periods. The results of this review indicate that multimorbidity increases both short- and long-term all-cause mortalities. This is consistent with previous findings that pre-existing or newly acquired comorbid conditions were more likely to be the main contributor to hospital readmission rates or death than HF exacerbation [3, 8, 65]. The results of our review may provide evidence that those who have survived acute heart conditions and are discharged from hospital settings may struggle to manage HF together with coexisting conditions.

Very few guidelines articulate what chronic conditions to consider when providing care for HF patients. Although recent HF management guidelines by the American Heart Association/American College of Cardiology/Heart Failure Society of America (AHA/ACC/HFSA) have recognized the negative impact and the complexity of managing comorbidity, they do not clearly outline their effects on patient outcomes (66). Rushton’s meta-analysis found DM, COPD, and renal dysfunction to be associated with all-cause mortality [6]. Similarly, DM and COPD were only conditions that are included in the Meta-analysis Global Group in Chronic Health Failure (MAGGIC), a relatively recently developed multivariable risk score to predict mortality in HF patients [67]. The developers of the MAGGIC risk scores have examined 30 cohort studies with a total of 39,372 HF patients for prognostic effects and reported that only DM and COPD were consistently included as comorbidity [67]. They also pointed out that of the 30 large cohort studies, the presence of DM was not assessed in one study and COPD was not assessed in 10 studies. In other words, even large-scale HF cohort studies may have overlooked some of the most important prognostic comorbidities.

On the other hand, we identified 32 individual conditions among HF patients from the included studies, and we were able to perform meta-analysis on 16 conditions. Our review found that DM, COPD, CKD, stroke, IHD, anemia, cancer, atrial fibrillation, dementia, depression, arrhythmia and arthritis to be statistically associated with higher mortality and hospital readmission rates in patients with HF. A previous narrative review also mentioned individual comorbid conditions that are associated with poor health outcomes in HF patients [5]. These conditions include all 16 conditions that were identified in the included studies. Although their narrative review also suggested conditions that were not included in our meta-analysis (e.g., thyroid and sleep disorders), they did not provide any quantitative results. It should be noted that although we identified 32 comorbid conditions, a meta-analysis could not be conducted for all conditions due to the lack of sufficient data. For example, only one of the included studies has study examined the effects of thyroid disorders and sleep apnea [52]. Our results suggest that a variety of comorbidities have yet to be investigated when examining the relationship between health outcomes and comorbidities. Also, the results of previous studies and those in our study indicate that among various comorbidities, DM, COPD, and renal dysfunction, especially CKD, may be the most important comorbidities to consider in relation to the prognosis of patients with HF.

Despite the importance of addressing multimorbid conditions in HF patients, this population has been either largely excluded or underrepresented from the most pivotal clinical trials and treatments [4, 5]. Contributing conditions have also been overlooked by researchers and clinicians when caring for the HF population even though this population puts considerable pressure on and presents significant challenges for cost-effective management. Even in some of our included studies, patients were excluded if they were diagnosed with chronic conditions or were receiving treatment for chronic conditions such as CKD [26], thyroid conditions [21], respiratory conditions [44] or psychological conditions [23]. Considering their negative effects on patient health outcomes, management of chronic conditions should be considered when providing care for HF patients.

Having multiple conditions can be a challenge for patients. Because medical systems are often fragmented, patients may have trouble comprehending and adhering to a variety of therapeutic regimens for each condition in a coherent manner. Intervention studies providing care coordination and/or supporting self-care have been effective to reduce the use of health services by patients with multi-morbidities [68, 69]. The need to address effective management of multimorbidity is critical, especially considering that the majority of the HF patients suffer from two or more coexisting chronic conditions.

Several limitations should be taken into consideration. Due to the lack of information in some of the included articles, we could not perform quantitative analyses on all studies due to lack of information. Therefore, some quantitative analyses were conducted based on only two studies, which may have led to heterogeneity between studies. In addition, our summary analyses included substantial heterogeneity, largely due to the observational study design of the included articles. Although the studies were mostly cohort studies with large sample sizes, studies with relatively small sample sizes were also included in the analyses. Although we confirmed a positive correlation between eight chronic conditions and poorer outcomes, it should be noted that this result may be underestimated. For example, COPD in HF is often underdiagnosed because its symptoms are similar to that of HF [9]. Lastly, eligible studies may have been excluded partly due to our inclusion criteria of selecting only publications written in English.

## Conclusion

Comorbidities, including cardiovascular and non-cardiovascular related conditions, are very common among HF patients. Healthcare providers must address and provide comprehensive assessment and management of HF including comorbid conditions that negatively affect HF outcomes. Patients with HF must be screened for comorbidities, especially DM, COPD, CKD, stroke, IHD, anemia, cancer, atrial fibrillation, dementia and depression, given that these conditions are likely to increase the risk of both mortality and hospital readmissions. Prompt and aggressive assessment, diagnosis, treatment, and coordinated care plans for these comorbidities should be reinforced to promote positive prognosis and patient-reported outcomes in the HF population.

### Electronic supplementary material

Below is the link to the electronic supplementary material.

Supplementary Material 1

## Data Availability

The data that support the findings of the study are available from individual included study, and the datasets analyzed during the current study are available from the corresponding author on reasonable request.

## List of Abbreviations

HF: Heart failure
PRISMA: The preferred items for systematic reviews and meta-analyses
RoBANS: The risk of bias assessment for non-randomized studies
HR: Hazard ratio
OR: Odds ratio
CI: Confidence interval
HRQoL: Health-related quality of life
DM: Diabetes mellitus
COPD: Chronic obstructive pulmonary disease
CKD: Chronic kidney disease
HTN: Hypertension
IHD: Ischemic heart disease
KCCQ: Kansas City Cardiomyopathy Questionnaire
SCHFI: Self-care of Heart Failure Index
SMD: Standardized mean difference
AHA/ACC/HFSA: American Heart Association/American College of Cardiology/Heart Failure Society of America
MAGGIC: Meta-analysis Global Group in Chronic Health Failure
