# Supplementary material for: Relationship between comorbidity and health outcomes in patients with heart failure: a systematic review and meta-analysis
Source: BMC Cardiovasc Disord. 2023 Oct 10;23:498. doi: 10.1186/s12872-023-03527-x (PMC10563307; doi:10.1186/s12872-023-03527-x)
Supplement: Supplementary file 1 — Supplementary Material 1 [file 12872_2023_3527_MOESM1_ESM.docx]

**Supplemental Material**

**Table S1.** General characteristics of included studies

| Last name of first author  (Year) | Total N  (% female) | Mean age | % of NYHA class III/IV | % LVEF <40%  (mean EF) | Follow-up period | HF definition | Comorbidity definition | Outcome measures | Recruitment setting |
| --- | --- | --- | --- | --- | --- | --- | --- | --- | --- |
| Adams (2012) | 985 (37.8) | 69 | 32.7 | NR (30.5) | 12 years | Clinical criteria | MI  DM | Mortality (all-cause) | Inpatient |
| Agrinier (2017) | 352 (24.1) | 65 | 100 | NR (22.3) | 15 years | Clinical criteria | HTN  DM  HL  CKD | Mortality (all-cause) | Inpatient |
| Ausili (2016) | 1,192 (41.8) | 72 | 40.8 | NR (44.6) | NR | Unspecified | HTN  DM  CKD | Self-care (SCHFI) | Outpatient |
| Bannay (2018) | 245 (31.0) | 65 | NR | 60 (34) | 1 year | Unspecified | DM  CAD | Readmission (HF-related) | Inpatient |
| Bektas (2017) | 186 (31.0) | 76 | 21 | NR (NR) | 2 years | Clinical criteria | COPD | Mortality (all-cause) | Outpatient |
| Bottle (2014) | 70,108 (49.5) | 78 | NR | NR (NR) | 30-days | Diagnosis code | Stroke  Pneumonia  IHD  Dementia  Arrythmia  HVD  PVD  HTN  COPD  CKD  Obesity  Any mental health (except dementia) | Readmission (HF-related)  Readmission (Non-HF-related) | Inpatient and Outpatient |
| Bottle (2019) | 6,360 (44.5) | NR^*^ | NR | NR (NR) | 1 year | Diagnosis code | Per extra comorbidity | Readmission (all-cause) | Outpatient |
| Castro (2021) | 463 (70.0) | 61 | NR | 100 (27) | 1.9 years | Clinical criteria | CKD  Stroke  Cancer  HTN  AF  DM  DL  Depression | Mortality (in-hospital) | Inpatient |
| Chamberlain (2017) | 121 (50.4) | 71 | NR | 49.6 (41.9) | NR | Clinical criteria | CCI | Self-care (SCHFI) | Inpatient |
| Chaudhry (2013) | 758 (50.5) | 80 | 30.3 | NR (NR) | 20 years | Clinical criteria | Obesity  CAD  CKD  DM  Stroke  COPD  Anemia | Readmission (all-cause) | Outpatient |
| Comin-Colet (2016) | 1,037 (30.1) | 71 | 45.1 | 33.7 (NR) | NR | Clinical criteria | CCI | HRQoL (KCCQ, EQ-5D, EQ-VAS) | Outpatient |
| Drozd (2021) | 1,794 (26.9) | 70 | 30.7 | NR (32) | 5 years | Clinical criteria | DM  COPD  CKD | Mortality (all-cause) | Outpatient |
| Ebner (2016) | 331 (17.0) | 64 | NR | 76 (34.7) | 2 years | Clinical criteria | Obesity | Mortality (all-cause) | Outpatient |
| Echouffo-Tcheugui (2016) | 364,480 (48.7) | 75 | NR | NR (NR) | 1 year | Diagnosis code | DM | Mortality (in-hospital)  Hospital LOS >4 days | Inpatient |
| Franco (2019) | 1,105 (52) | 79 | 25 | NR (21%) | 1 year | Clinical criteria | CCI | Mortality (all-cause) | Inpatient |
| Huang (2017) | 129 (57.4) | 75 | NR | 52 (NR) | 3.1 years | Clinical criteria | CCI | Mortality (all-cause) | Inpatient |
| Khafaji (2015) | 5,005 (37.4) | 60 | 75.2 | 36.96 (NR) | 1 year | Clinical criteria | DM  CKD  COPD/asthma  STEMI  HVD  Stroke  PVD | Mortality (in-hospital)  Mortality (all-cause) | Inpatient |
| Korda (2017) | 5,074 (42.4) | 80 | NR | NR (NR) | 30 days | Diagnosis code | CVD  COPD  Anemia  Dementia  CCI | Mortality (all-cause)  Readmission (all-cause) | Inpatient |
| Lawson (2021) | 698,983 (49.7) | NR^*^ | NR | NR (NR) | 30 days | Diagnosis code | HTN  AF  DM  COPD  Asthma  Depression  Cancer  CKD  CVA  Dementia  Anemia  Arthrosis  Rheumatoid arthritis | Readmission (all-cause)  Readmission (HF-related)  Readmission (Non-HF-related) | Inpatient |
| Le Corvoisier (2014) | 555 (66.1) | 85 | NR | NR (NR) | Until discharge or death | Clinical criteria | CVD | Mortality (in-hospital) | Inpatient |
| Lee (2017) | 580 (41.0) | 66 | 34 | NR (NR) | 2 years | Unspecified | CCI | Self-care (EHFScB-9) | Inpatient and Outpatient |
| Manemann (2016) | 1,714 (56.2) | 76 | NR | NR (NR) | 4.2 years | Multiple | CAD  Arrhythmia  Stroke  HTN  HL  DM  Arthritis  Osteoporosis  Asthma  COPD  CKD  Cancer  Depression  Dementia  Schizophrenia  Substance abuse | Mortality (all-cause)  Readmission (all-cause) | Inpatient and Outpatient |
| Matsuoka (2016) | 227 (37.4) | 68 | 4.8 | 32.2 (46.5) | NR | Unspecified | HTN  CKD  AF  DM | Self-care (EHFScB-9) | Outpatient |
| Maymon (2021) | 8,332 (49.5) | NR^*^ | NR | NR (NR) | 30 days | Diagnosis code | DM  HTN  IHD  COPD  AF  Renal Failure  Anemia  Valvular disease  Obesity  MI  PVD | Readmission (all-cause) | Inpatient |
| Mulla (2021) | 721 (46.0) | 71 | 21 | NR (40) | 1 year &  10 years | Multiple | CCI  DM  HTN  DL  COPD  PVD  AF/A-Flutter  IHD  MI  CVA | Mortality (all-cause) | Inpatient |
| Munoz-Rivas (2019) | 1,501,811 (54.9) | 79 | NR | NR (NR) | 5-years | Diagnosis code | DM  CCI | Mortality (in-hospital)  Readmission (all-cause)  Hospital LOS >4 days | Inpatient |
| Nayar (2018) | 4,319 (49.5) | 77 | NR | NR (NR) | NR | Diagnosis code | CCI | Mortality (in-hospital) | Inpatient |
| Ogah (2014) | 262 (47.3) | 56 | 92.4 | NR (39.7) | 6 months | Clinical criteria | AF  DM | Readmission (all-cause) | Inpatient |
| Omersa (2016) | 34,406 (54.8) | 78 | NR | NR (NR) | 1 year | Diagnosis code | HTN  AF  MI  IHD  DM  CKD  COPD  Cancer | Readmission (HF-related) | Inpatient |
| Park (2021) | 3,818 (47.2) | 71 | 41.8 | 53.3 (40.2) | 7 years | Clinical criteria | AF  HTN  DM  IHD | Mortality (all-cause) | Inpatient |
| Sharma (2018) | 73,878 (54.4) | NR^*^ | NR | NR (NR) | 30 days | Unspecified | Per extra non-cardiovascular comorbid | Mortality (all-cause)  Readmission (all-cause)  Readmission (HF-related) | Inpatient |
| Sokoreli (2018) | 779 (35.0) | NR^*^ | 90 | 42 (NR) | 1 year | Clinical criteria | DM  MI  COPD  Cancer | Readmission (all-cause) | Inpatient |
| Streng (2018) | 3,499 (29.7) | 71 | 43.6 | NR (66) | 2.1 years | Multiple | DM  CKD  COPD  Stroke  Thyroid problem  Anemia  Obesity  PAD | HRQoL(KCCQ) | Inpatient and Outpatient |
| Sze (2021) | 467 (33.0) | NR^*^ | 22.0 | NR (NR) | 1.5 years | Clinical criteria | CCI  MI  PVD  HTN  CVA  DM  Dementia  COPD  Depression  Anemia  Urinary incontinence | Mortality (all-cause) | Outpatient |
| Targher (2017) | 6,926 (37.0) | 69 | 84.9 | NR (39.2) | 1 year | Clinical criteria | DM  HTN  Stroke  COPD  Obesity  CKD | Mortality (in-hospital)  Mortality (all-cause)  Readmission (all-cause) | Inpatient and Outpatient |
| Van den Berge (2021) | 334 (34.0) | NR^*^ | 40 | NR (NR) | 1 year | Clinical criteria | HTN  AF  DM  COPD  CKD  CVA | HRQoL (KCCQ, EQ-5D, EQ-VAS) | Inpatient |
| Van Deursen (2014) | 3,226 (39.7) | 66 | 28.2 | 49.98 (NR) | 11.7 months | Clinical criteria | CKD  Anemia  DM  COPD  Stroke  Sleep apnea  Hypothyroidism  Hyperthyroidism | Mortality (all-cause)  Readmission (HF-related) | Outpatient |
| Voors (2017) | 2,516 (26.6) | 69 | 60.5 | 31 (NR) | 1.8 years | Clinical criteria | DM  COPD | Mortality (all-cause)  Readmission (all-cause) | Inpatient and Outpatient |
| Wakabayashi (2017) | 4,842 (42.1) | 73 | 81.4 | NR (53.3) | NR | Clinical criteria | COPD | Mortality (in-hospital) | Inpatient |
| Wienbergen (2018) | 949 (25.3) | 70 | 70.5 | NR (NR) | 1 year | Unspecified | Anemia  COPD  Cancer | Mortality (all-cause) | Inpatient and Outpatient |
| Wray (2021) | 1,500 (NR) | 78 | NR | NR (NR) | 30 days | Diagnosis code | Acute coronary syndrome  Coronary atherosclerosis  HVD  Arrhythmia  Other unspecified heart disease  Stroke  CKD  COPD  DM  Dementia  Cancer  Chronic liver disease  Severe hematologic disorders  Iron deficiency  Depression  Chronic lung disorder  Asthma  Nephritis  Urinary tract disorders  Other psychiatric disorders  Peptic ulcer and other Specified GI tract disorders  Other GI tract disorders  Decubitus skin ulcer | Readmission (all-cause) | Inpatient |
| Zaharova (2021) | 73 (49.3) | 65 | 53.4 | NR (NR) | NR | Clinical criteria | CCI | Self-care (SCHFI) | Outpatient |

Note. Studies are listed in alphabetical order. ^*^ mean age not reported; AF, atrial fibrillation; CAD, coronary artery disease; CCI, Charlson Comorbidity Index; CKD, chronic kidney disease; COPD, chronic obstructive pulmonary disease; CVA, cerebrovascular accident; CVD, cerebrovascular disease; DL, dyslipidemia; DM, diabetes mellitus; EHFScB-9, 9-item European Heart Failure Self-care Behavior Scale; EQ-5D, EuroQoL-5 Dimension; EQ-VAS, EuroQoL Visual Analog; HF, heart failure; HL, hyperlipidemia; HRQoL, health-related quality of life; HTN, hypertension; HVD, heart valve disease; IHD, ischemic heart disease; KCCQ, Kansas City Cardiomyopathy Questionnaire; LOS, length of stay;  LVEF, left ventricular ejection fraction; MI, myocardial infarction; NR, not reported; NYHA, New York Heart Association; PAD, peripheral artery disease; PVD, peripheral vascular disease; SCHFI, Self-Care of Heart Failure Index; STEMI, ST segment elevation myocardial infarction


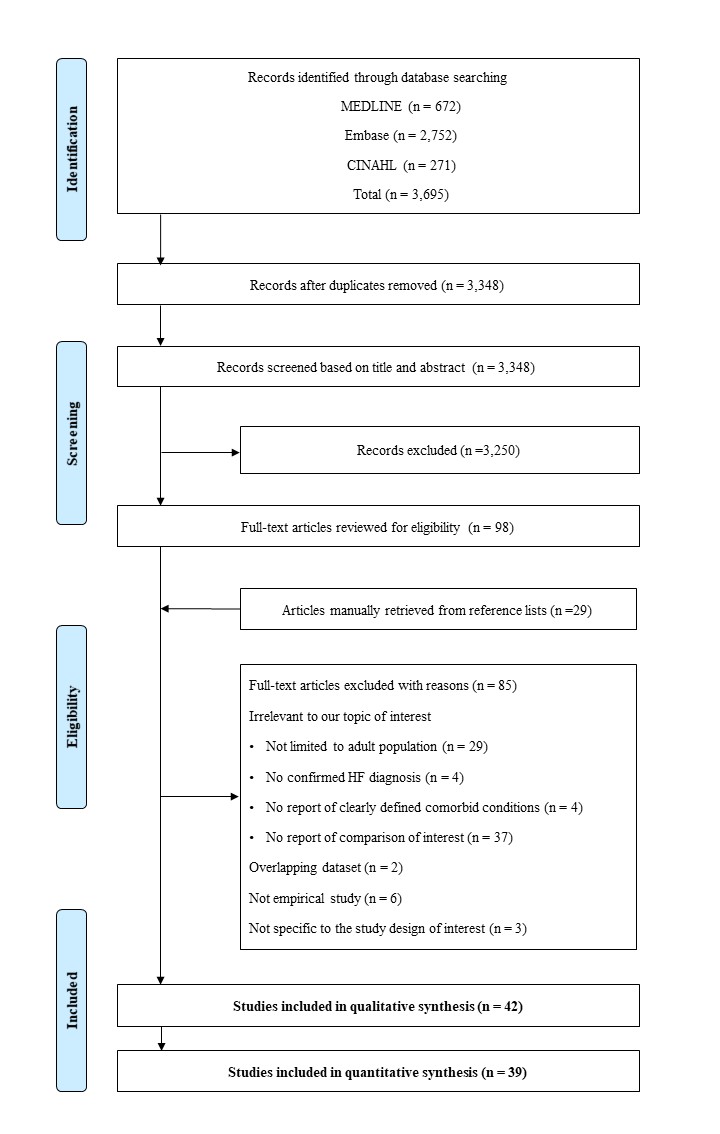


**Figure S1.** The flow diagram of literature search and selection process


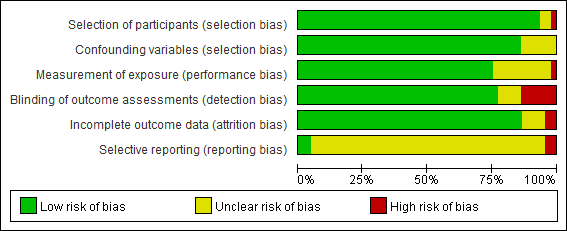


**Figure S2.** The summary of risk of bias

**
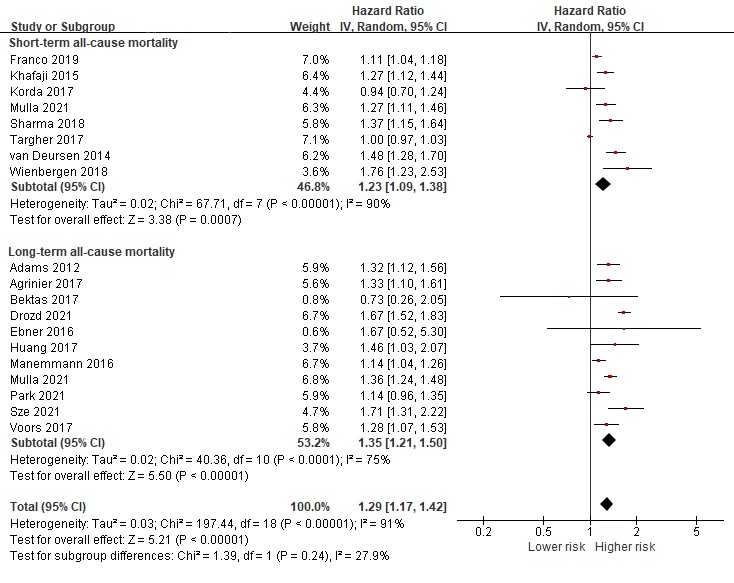
**

**Figure S3.** Forest plot of the pooled analysis evaluating the effect of comorbidities on all-cause mortality based on follow-up period


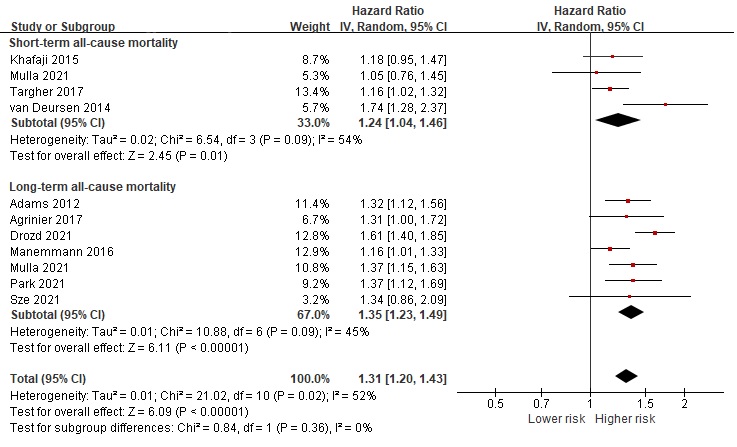


**Figure S4.** Forest plot of the pooled analysis evaluating the effect of diabetes mellitus on all-cause mortality in heart failure patients based on follow-up period


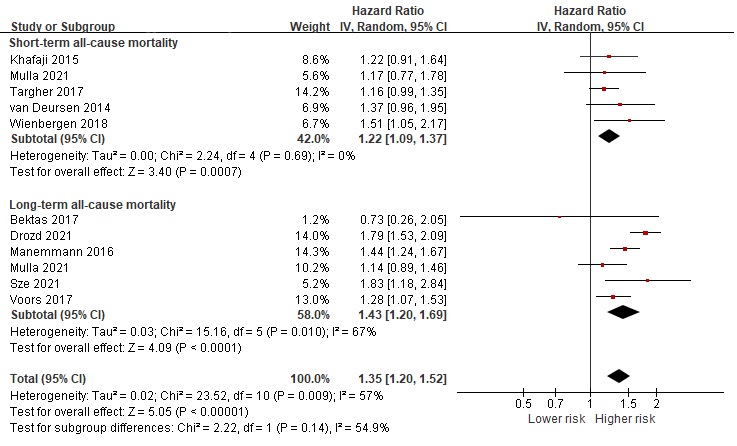


**Figure S5.** Forest plot of the pooled analysis evaluating the effect of chronic obstructive pulmonary disease on all-cause mortality in heart failure patients based on follow-up period


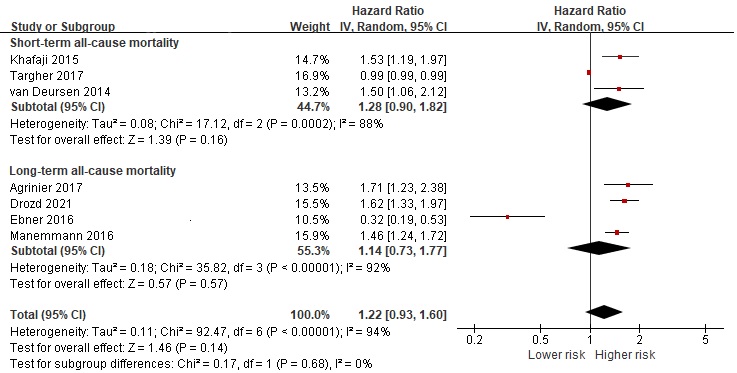


**Figure S6.** Forest plot of the pooled analysis evaluating the effect of chronic kidney disease on all-cause mortality in heart failure patients based on follow-up period


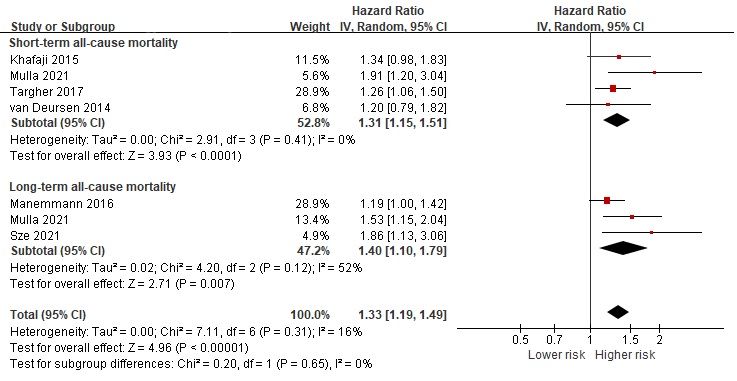


**Figure S7.** Forest plot of the pooled analysis evaluating the effect of stroke on all-cause mortality in heart failure patients based on follow-up period


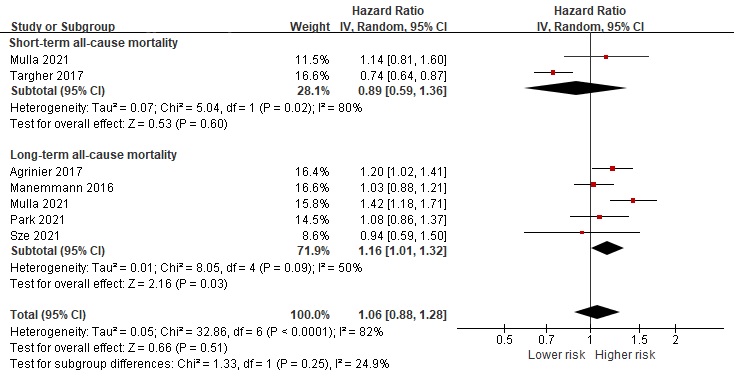


**Figure S8.** Forest plot of the pooled analysis evaluating the effect of hypertension on all-cause mortality in heart failure patients based on follow-up period


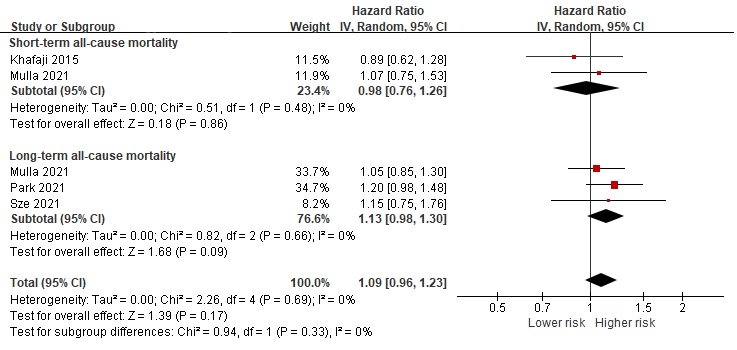


**Figure S9.** Forest plot of the pooled analysis evaluating the effect of ischemic heart disease on all-cause mortality in heart failure patients based on follow-up period


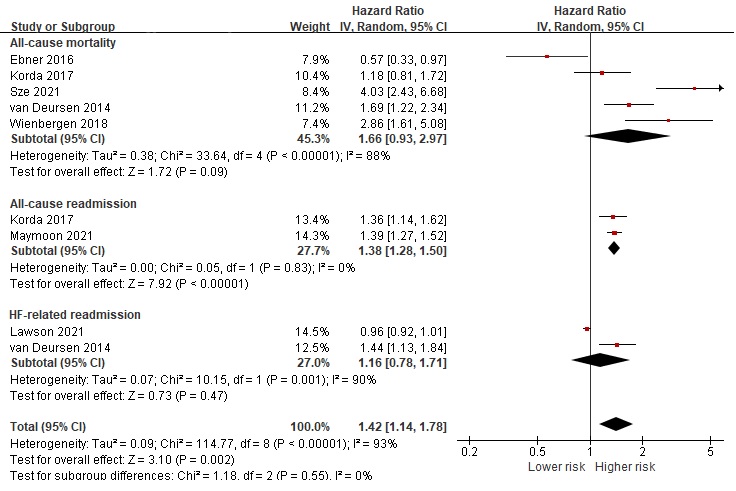


**Figure S10.** Forest plot of the pooled analysis evaluating the effect of anemia on health outcomes in heart failure patients


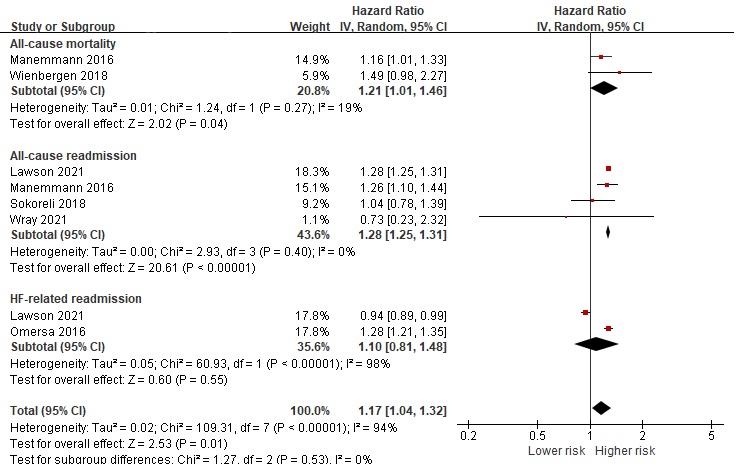


**Figure S11.** Forest plot of the pooled analysis evaluating the effect of cancer on health outcomes in heart failure patients


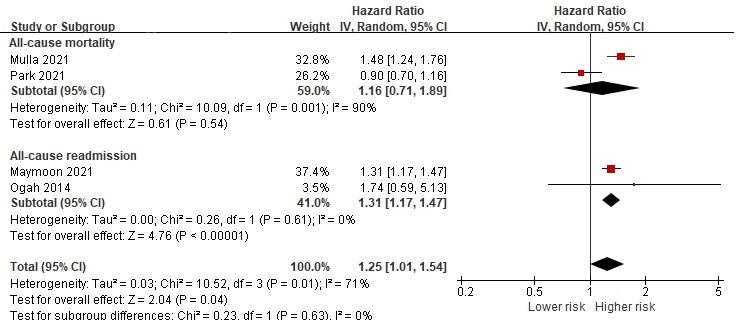


**Figure S12.** Forest plot of the pooled analysis evaluating the effect of atrial fibrillation on health outcomes in heart failure patients


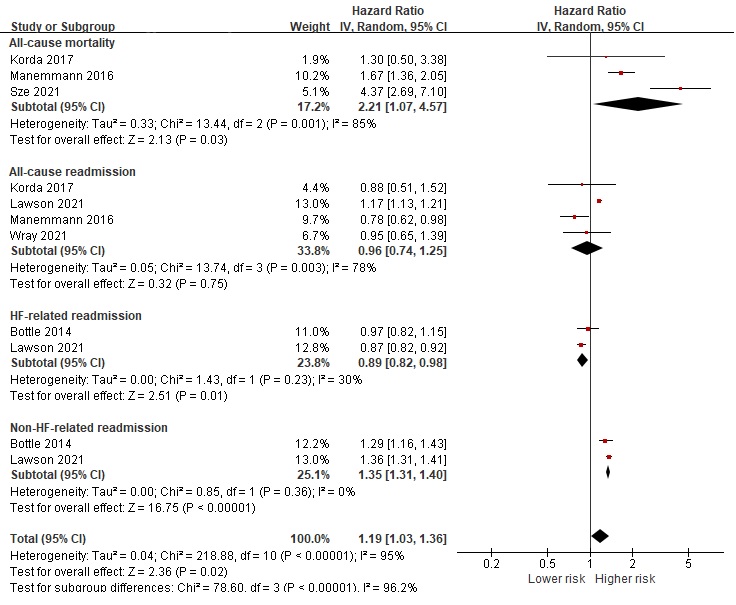


**Figure S13.** Forest plot of the pooled analysis evaluating the effect of dementia on health outcomes in heart failure patients


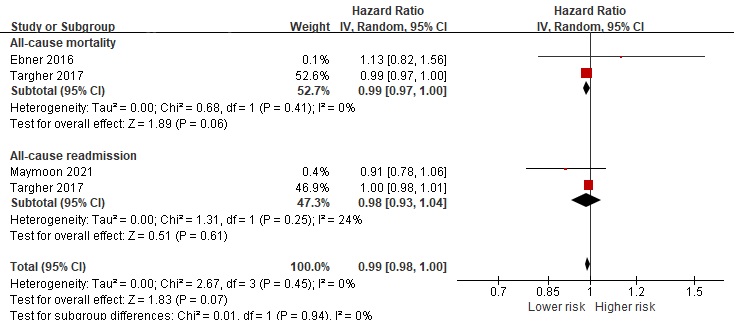


**Figure S14.** Forest plot of the pooled analysis evaluating the effect of obesity on health outcomes in heart failure patients


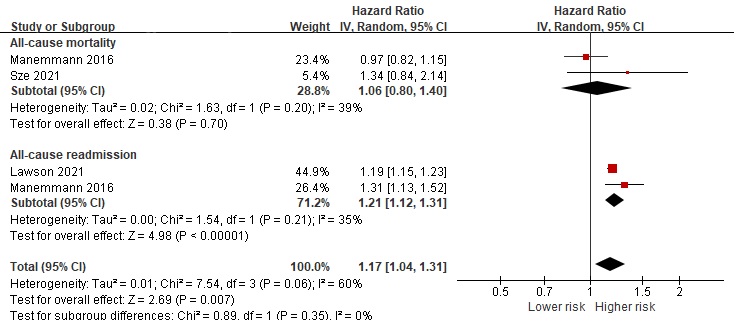


**Figure S15.** Forest plot of the pooled analysis evaluating the effect of depression on health outcomes in heart failure patients


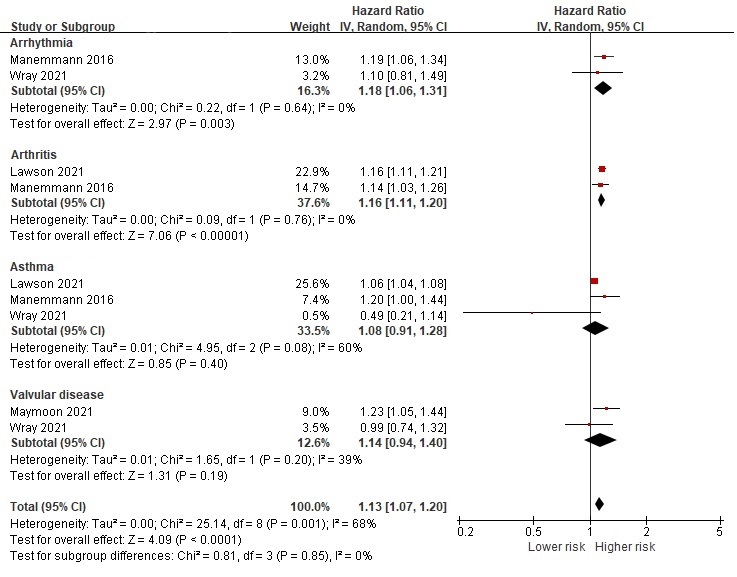


**Figure S16.** Forest plot of the pooled analysis evaluating the effect of arrhythmia, arthritis, asthma and valvular disease on all-cause readmission in heart failure patients
